# On Kantian tendencies during the early corona pandemic in Germany

**DOI:** 10.1007/s11299-020-00272-y

**Published:** 2020-12-23

**Authors:** Markus A. Feufel, Christine Schmid, Viola Westfal

**Affiliations:** Division of Ergonomics, Department of Psychology and Ergonomics (IPA), Technische Universität Berlin, Straße des 17. Juni 135, 10623 Berlin, Germany

**Keywords:** Risk perception, Preventive behaviours, Coronavirus, SARS-CoV-2, Covid-19 pandemic, Policy making

## Abstract

Based on an ad hoc online survey about risk perception and preventive behaviours, we describe three chronological phases related to how people in Germany perceived the Corona pandemic between March 22 and May 10, 2020. In general, participants reported to be less concerned about their own risk than about the risk faced by others. However, a good portion of those who thought that they themselves were low risk actually wrote about their belief that they nevertheless had a responsibility to behave in ways that benefited others, even if it came at a cost to themselves. In loose reference to Immanuel Kant’s notion that humans have a rational duty to act in a socially responsible manner, we interpret people’s comments about other-regarding behaviour as an initiation of a Kantian tendency during the Corona pandemic. Based on these findings, we suggest that policy makers may do better in times of crisis than nudging, incentivizing, or compelling the public by law. They can perhaps accomplish more by (also) nurturing people’s innate sense of the need for socially responsible action to be taken in order to meet the daunting challenges of present and future crises.

## Introduction

Between March 22 and May 10, 2020, we collected about 2800 (non-representative) data points on risk perception and self-reported preventive behaviours during the lockdown that was in place in Germany due to the Corona pandemic. We also collected about 1100 responses to an open-ended question ("What is currently on your mind?"), with answers reflecting participants’ thoughts and reasoning during the same time frame. In general, participants were less concerned about their own risk than they were about the risk that their fellow citizens faced. Rather than focusing mainly on their own personal, economic, and emotional situations, many participants wrote about their perceived duty to behave in ways that benefited others, even if it involved some personal sacrifice, and reported adherence to preventive behaviours. In loose reference to Immanuel Kant’s notion that—independent of any personal benefit—humans have a rational duty to act in a socially responsible manner, we interpret people’s own self-reported behaviours and their comments about other-regarding behaviour as an expression of Kantian tendencies during the Corona pandemic.^1^ Of course, since our data are non-representative, we cannot derive definitive conclusions from them, so our contribution should be read as an empirically informed opinion aimed at stimulating discussion. In the following, we will first outline the three phases of risk perception identified during the early pandemic and then use the open-ended question responses to shed light on how people in our sample reacted to the risks they perceived and the Kantian tendencies they demonstrated. We conclude with implications for policy makers.

## Risk perception and self-reported behaviours

**Fig. 1:**
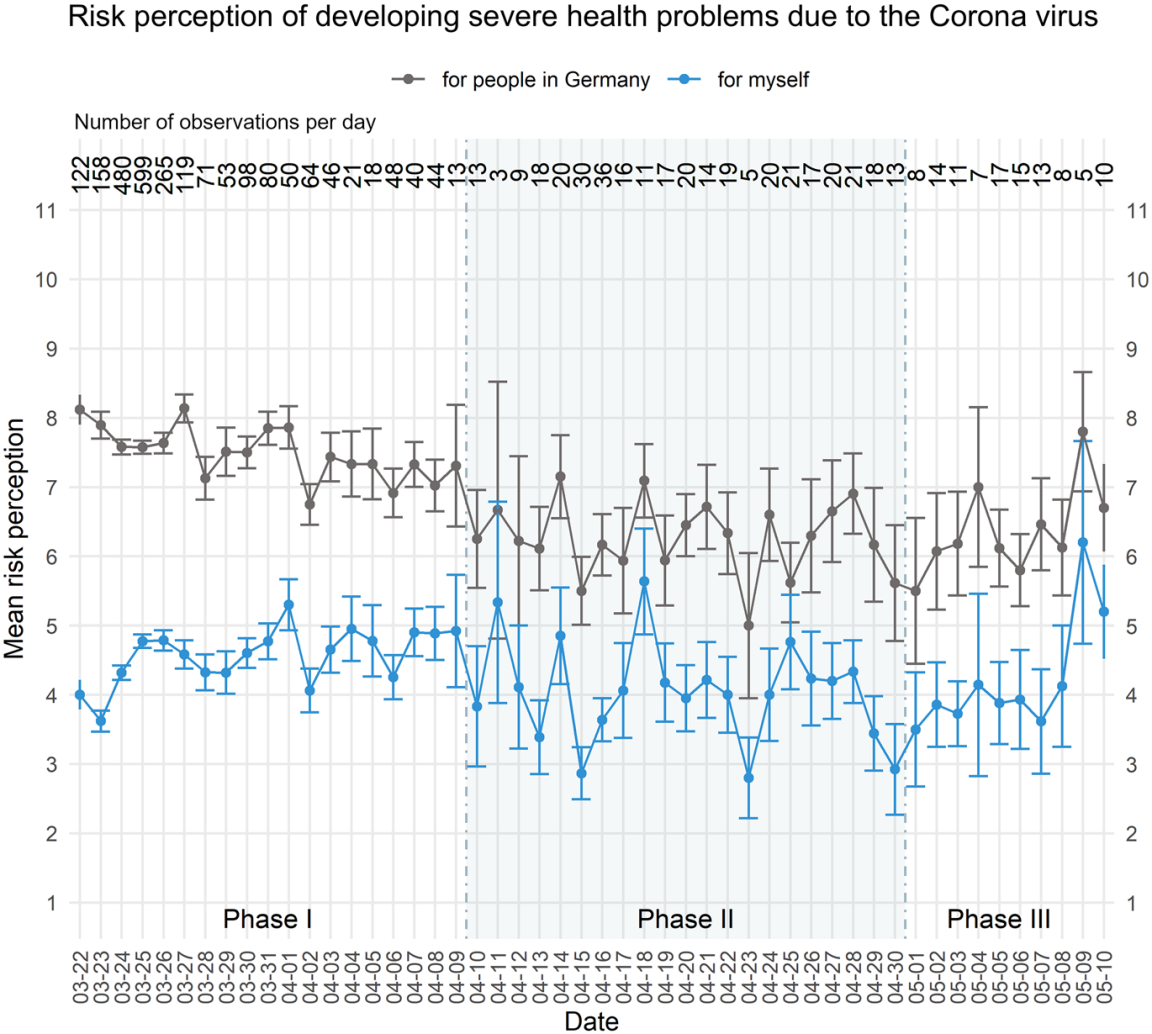
Participants’ mean ratings (error bars provide the standard error) per day on a 10-point Likert scale (1—extremely low to 11—extremely high) concerning the questions “How do you currently perceive the *risk for people in Germany* of developing severe health problems from the virus?” (gray) and “How do you currently perceive *your own risk* of developing severe health problems from the virus?” (blue). Overall, N = 2859 observations; the number of observations per day is given at the top of the figure. Three phases with characteristic patterns in the data are separated by a shaded background

There have been several representative surveys assessing changes in people's risk perception, fear, and coping strategies during the Corona pandemic in Germany (Betsch et al. 2020; Blom et al. 2020; Bundesinstitut fuer Risikobewertung 2020; Gerhold 2020; for an overview of research projects on Covid-19 in Germany see: *Forschung zur Corona-Pandemie | RatSWD—Rat für Sozial- und Wirtschaftsdaten*). Our data, by contrast, are based on an ad hoc, non-representative online survey that could be taken by the same person as often as desired and at a time of their own choosing. The average age of participants was 37.2 years (SD = 13.7, min = 16, max = 91), which is lower than the average age of the general population in Germany (M = 44.5) (Statista 2020), a fact that may in part account for the relatively low risk that the participants perceived for themselves as compared to the risk they perceived for others (see Fig. 1). Because our data are non-representative, we cannot evaluate risk perceptions and behaviours in any meaningful way in relation to the actual risks present at the time. However, the combination of answers to both closed- and open-ended questions allows us to supplement existing studies by documenting how some of the people in our sample reacted to the risks they perceived as well as the Kantian tendencies they demonstrated during the early Corona pandemic.

Figure 1 displays the mean perceived risk of developing severe health problems from the virus as an *individual* (in blue) and in general *for people in Germany* (in gray) for each day of data collection. Following studies that captured similar patterns and trends of risk perception in representative samples (Blom et al. 2020), in our data we distinguish three phases and document participants’ responses with respect to those phases: (I) a relatively high and stable risk perception at the outset (March 22-April 9), (II) an intermediate phase with a slowly decreasing risk perception (April 10–April 30), and (III) a phase characterized by the risk perception rising once again (May 1–May 10). Although the designation of these phases is supported by representative studies of risk perception in Germany, we do not suggest that the boundaries between these phases are clear-cut. Instead, we use the phases pragmatically to help outline trends in how people’s risk perception, behaviour, and reaction to perceived risks changed over the early course of the pandemic.

**Fig. 2:**
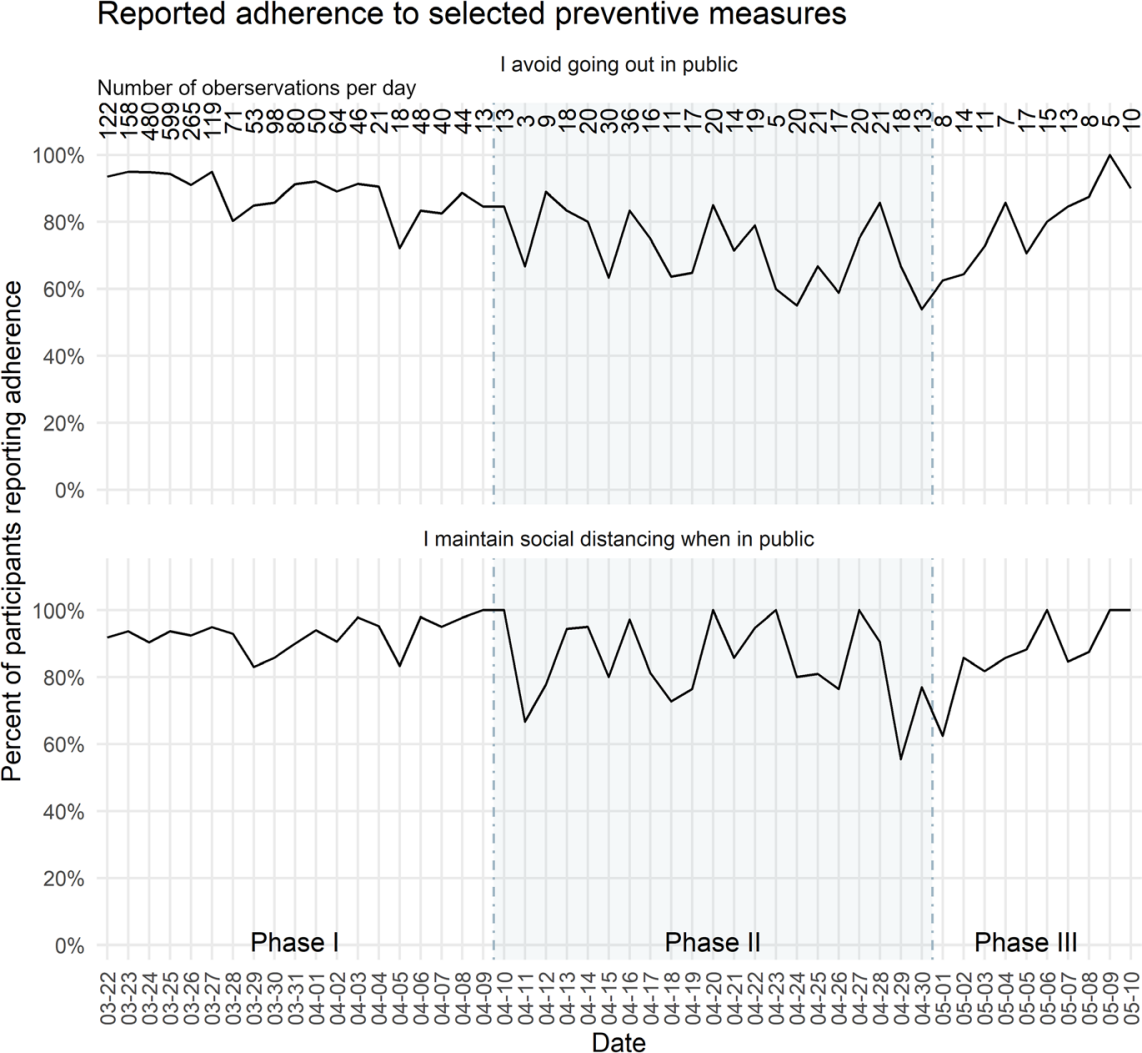
Proportion in percentage of participants adhering to selected preventive measures between March 22 and May 10, 2020. Overall, N = 2859 observations; the number of observations per day is given at the top of the figure. Three phases with characteristic patterns in the data on risk perception are separated by a shaded background

Figure 2 shows that the self-reported adherence to prescribed preventive behaviours resulted in similar though less pronounced patterns: compared to the data on risk perception, a relatively high and stable proportion of participants reported avoiding going out in public, as well as maintaining social distancing when in public across all three phases, with only minor decrements in self-reported behaviours during phase II. In the following section, we use the answers to the open-ended question to discuss how the people in our sample reacted to the risks that they perceived.

## When and what kind of Kantian tendencies occurred

Phase I (March 22–April 9; 19 days).

I care for my parents and children (…) I try to do everything at once when I go shopping, so that I do not endanger the lives of others by spreading Corona. (March 25, 2020).

The plight of people to whom the situation cannot be explained (people with mental disabilities and with dementia), and of those nobody speaks to: the homeless and drug-addicted people, sex workers… (March 23, 2020).

The initial phase of the lockdown in Germany was marked by strict rules enforced by the government and the relatively high risk that people perceived, both for themselves and, to an even greater degree, for others. During this phase 2389 people participated, 883 (37%) of whom made an open-ended comment about what was on their mind at the time. Although most of those participants (603/883, 68%) commented about their personal emotional reactions to the pandemic, their economic situations, and changes to the course of their everyday lives, 280 (32%) of them reported a sense of a social responsibility to behave in ways that would protect, help, or support others. For instance, participants worried about people who were worse off than they were and wrote about their responsibility to act in ways that might protect their fellow human beings, or about their humanitarian duty to help those in need. Whereas most of these comments reflected participants’ readiness to act in a socially responsible way or the fact that positive social developments were already taking place in their social circles (e.g., increasing social cohesion, solidarity), 66 of the 280 comments (24%) stated that they engaged in socially responsible behaviour in order to compensate for fellow citizens who might not live up to the same standard. In other words, at the beginning of the pandemic, participants demonstrated not only concern for their own situation but also for that of others, and a willingness to act responsibly, even if that involved a cost (e.g., replanning of grocery shopping, going out of their way for others who might not appreciate their sacrifice) to themselves.

Phase II (April 10–April 30; 21 days).

Currently, I am experiencing how the restrictions are increasingly bothering me, even though I consider them necessary and important. (April 13, 2020).

Uncertainty and fear of the medium-term future—my employment contract will probably be terminated by the end of May. (April 17, 2020).

After several weeks in a state of lockdown, risk perception, reported adherence to protective behaviors, and the number of participants decreased. Of the 361 participants during that phase, 151 (42%) made an open-ended comment. Of those 151 comments, 37 (24%) referred to Kantian tendencies, that is, a perceived duty to behave in ways that benefit others, in particular marginalized risk groups. In 16 of the 37 comments (43%) participants reported that they had to act in a socially responsible way in order to compensate for those who were not assuming the same level of social responsibility. All other comments suggested that around Easter (April 10), most participants started to scrutinize the Corona virus as the main topic dominating both public and personal discourse. With reference to the relatively milder spread of the virus at the time (infection numbers decreased during this period in Germany), the measures being used to fight the virus became subject to debate, and questions about possible social and economic consequences of the lockdown and how to avoid them were raised. Participants also reported concern about other topics, for instance, Australian bushfires, and their own economic, psychological, and social needs and worries (e.g., people wanted to celebrate Easter with their families rather than follow health guidelines). In sum, although most comments tended to focus on personal emotional reactions and economic concerns, more than 20% still focused on other-regarding behaviors and ways to address others’ needs.

Phase III (May 1–May 10; 10 days).

I see that the virus is increasingly dealt with carelessly in everyday life (…) as if one could behave as before without second thoughts. (May 5, 2020).

I’m annoyed that at times many people seem to forget that the corona problem still exists and do not keep a distance or wear face masks in public… when you do, people look at you like an alien and may even attack you verbally. (May 5, 2020).

In May, the lockdown measures were relaxed, and the number of participants decreased further (n = 109). At the same time, the perception of both personal risk and risk for other people in Germany started to rise again, as did the reported rate of preventive behaviours. Of the 109 participants, 52 (48%) made an open-ended comment. Of those 52 comments, 12 instances (23%) reflected Kantian tendencies. Based on respondents’ fears that regulations might have been relaxed prematurely, 8 of these 12 comments (67%) suggested that participants worried about others’ sense of responsibility, and that they perceived a duty to compensate for or counterbalance the behavior of those unwilling to conform to preventive behaviors. In other words, also during this phase about 20% of the participants were still concerned about others. Specifically, they not only worried about protecting the well-being of others and helping those in need, but, compared to the previous phases, an even higher percentage felt that they needed to counterbalance the behaviors of those who might lack the same sense of social responsibility and thereby cause a second wave of infections.

## Conclusion

Although most people participating in our survey perceived the Corona virus to pose a low risk to themselves, across all phases, a sizeable proportion of at least 50% of the participants reported adhering to protective behaviours and more than 20% mentioned a perceived social responsibility for their fellow citizens—in order to help those in need or at risk and, with increasing tendency in the later phases, also to compensate for those who lacked a similar sense of social responsibility. In our opinion, these findings suggest that a sizeable proportion of the German population was ready to put personal interests aside during a time of crisis and initiate duty-based Kantian tendencies. The three chronological phases that we captured until May 10 certainly do not allow for predictions to be made about how people will perceive risk and what they will think or do in the case of a second or third wave. But based on the Kantian tendencies we identified in this group at the outset of the pandemic, we suggest that policy makers may do better in times of crisis than nudging, incentivizing, or compelling the public by law. They can perhaps accomplish more if they build on the percentage of the public that already initiates Kantian tendencies by developing strategies that specifically nurture these tendencies and cultivate all people’s understanding of the kind of socially responsible action that will help to meet the daunting challenges of present and future crises.

## Footnotes

1 We chose the term "tendencies" because we do not want to equate people’s situation-specific responses to the definition of a *Kantian maxim* (which would entail that participants referred to subjective principles for rational action in general), let alone a *Kantian imperative* (which would require that participants suggested that they act only according to maxims that, if applied as a universal rule, would still be acceptable).
